# A retrospective study of two populations to test a simple rule for spirometry

**DOI:** 10.1186/s12875-016-0467-2

**Published:** 2016-06-04

**Authors:** Jill A. Ohar, Barbara P. Yawn, Gregg L. Ruppel, James F. Donohue

**Affiliations:** Department of Internal Medicine, Wake Forest School of Medicine, Medical Center Boulevard, Winston-Salem, NC 27157-1054 USA; Department of Research, Olmsted Medical Center, Rochester, MN 55904 USA; Pulmonary, Critical Care & Sleep Medicine, Saint Louis University School of Medicine, Saint Louis, MO USA; University of North Carolina Chapel Hill, Chapel Hill, NC USA

**Keywords:** COPD, Spirometry, Smoking, Respiratory symptoms

## Abstract

**Background:**

Chronic lung disease is common and often under-diagnosed.

**Methods:**

To test a simple rule for conducting spirometry we reviewed spirograms from two populations, occupational medicine evaluations (OME) conducted by Saint Louis and Wake Forest Universities at 3 sites (*n* = 3260, mean age 64.14 years, 95 % CI 58.94–69.34, 97 % men) and conducted by Wake Forest University preop clinic (POC) at one site (*n* = 845, mean age 62.10 years, 95 % CI 50.46–73.74, 57 % men). This retrospective review of database information that the first author collected prospectively identified rates, types, sensitivity, specificity and positive and negative predictive value for lung function abnormalities and associated mortality rate found when conducting spirometry based on the 20/40 rule (≥20 years of smoking in those aged ≥ 40 years) in the OME population. To determine the reproducibility of the 20/40 rule for conducting spirometry, the rule was applied to the POC population.

**Results:**

A lung function abnormality was found in 74 % of the OME population and 67 % of the POC population. Sensitivity of the rule was 85 % for an obstructive pattern and 77 % for any abnormality on spirometry. Positive and negative predictive values of the rule for a spirometric abnormality were 74 and 55 %, respectively. Patients with an obstructive pattern were at greater risk of coronary heart disease (odds ratio (OR) 1.39 [confidence interval (CI) 1.00–1.93] vs. normal) and death (hazard ratio (HR) 1.53, 95 % CI 1.20–1.84) than subjects with normal spirometry. Restricted spirometry patterns were also associated with greater risk of coronary disease (odds ratio (OR) 1.7 [CI 1.23–2.35]) and death (Hazard ratio 1.40, 95 % CI 1.08–1.72).

**Conclusions:**

Smokers (≥ 20 pack years) age ≥ 40 years are at an increased risk for lung function abnormalities and those abnormalities are associated with greater presence of coronary heart disease and increased all-cause mortality. Use of the 20/40 rule could provide a simple method to enhance selection of candidates for spirometry evaluation in the primary care setting.

## Background

Spirometry is an important tool for assessing lung function and the development of mobile, accurate and less expensive equipment has allowed the assessment of spirometry in primary care settings [1–3]. However, spirometry testing remains underutilized in primary care [4, 5], perhaps due to the lack of clarity of when to use spirometry evaluation. Universal spirometry screening has been assessed for improved Chronic Obstructive Pulmonary Disease (COPD) diagnoses and found to be too expensive, requiring testing of up to 465 individuals to deter one COPD exacerbation [6]. Guidance is required to select an appropriate subset of the general population in whom to complete spirometry testing.

But smokers are at increased risk for a variety of abnormalities of lung function, not just COPD and lung function abnormalities have been reported in more than half of current smokers [7]. Risk for, and under-detection of, pulmonary function abnormalities, increase with increasing age [8]. Pulmonary function abnormalities are markers for increased morbidity and mortality [8–15]. Despite this association pulmonary function abnormalities frequently go undetected in clinical practice [4, 16–18].

Selective use of spirometry has been directed primarily toward identification of individuals with undiagnosed COPD. For example, Global Initiative for Chronic Obstructive Lung Disease (GOLD) suggests spirometric testing for COPD identification based on age > 40 years and risks, such as smoking history, or symptoms [19]. The American College of Physicians recommends testing based on presence of both smoking history and symptoms, such as cough, dyspnea, or wheeze, for testing [20]. This requires collection of new clinical information that may not be in the existing medical records nor volunteered by the patient.

Conversely, all medical records should include the patient’s age and smoking status. We present data on the use of a simple rule that includes only the patient’s age and smoking history to determine the need for spirometry to detect lung abnormalities including those beyond the obstructive patterns most consistent with COPD. In addition, we assess the mortality risk associated with the identified lung abnormalities [8–13].

## Methods

### Study populations

The OME group included workers screened for occupation lung disease conducted by Saint Louis and Wake Forest Universities at 3 sites. Details of a portion of this population have been reported previously [21]. Criteria for occupational medical evaluation (OME) was occupational exposure to asbestos that was at least 10 years prior to evaluation and the possibility of an abnormal chest x-ray (i.e., the presence of pleural plaque or International Labor Union scoring of 0/1 or above). Data came from retrospective review of clinical data and pulmonary function testing acquired 1983–2010 that was housed in a data repository. Only subjects with a complete set of data were used. All individuals had a brief history, chest radiograph and pulmonary function testing. The history included asking subjects if a healthcare professional had ever given them a diagnosis of chronic obstructive pulmonary disease, asthma, or heart disease including angina, myocardial infarction or coronary heart disease. Subjects were included in the analysis regardless of whether they were previously diagnosed with COPD, asthma or not. Details of the reliability of symptoms and clinical diagnosis of COPD in a portion of this population have previously been published (21). Cigarette smoking history (never, former or current) was further quantified by pack-years and a chest radiograph was completed for each patient. Based on published chronic obstructive pulmonary disease demographics [22], enrollment was limited to individuals age ≥ 40 years without evidence of significant work related chest x-ray abnormalities. This protocol was reviewed and approved by the Saint Louis University and Wake Forest University Institutional Review Boards (IRB) prior to the initiation of the study.

To determine the reproducibility of the 20/40 rule for conducting spirometry, the rule was applied to a population of current or former smokers, 40 years of age and older, who had smoked 20 or more pack years, seen in the preoperative clinic (POC) before elective surgery, at Wake Forest University Baptist Medical Center. Subjects were evaluated with spirometry from October 2013 to July 2014.

### Pulmonary function testing

Pulmonary function testing was performed by respiratory therapists and a registered nurse, according to ATS guidelines [23]. Spirometers (a Collins CPL at the 2 Wake Forest OME sites, Medgraphics at the Wake Forest POC site and a Puritan Bennett Renaissance at the Saint Louis University OME site) were all calibrated daily. All forced vital capacity (FVC) maneuvers lasting less than six seconds were discarded. Spirometric data were expressed as percent predicted using National Health and Nutrition Examination Survey (NHANES) III predicted equations [24]. Airflow obstruction was defined as a ratio of pre-bronchodilator FEV_1_ / FVC < 70 % (forced expiratory volume in one second/forced vital capacity) derived from modification of National Institute for Clinical Excellence (NICE) [25] and Global Initiative for Chronic Obstructive Lung Disease (GOLD) recommendations necessary since post-bronchodilator testing was not available [26]. Spirometry was classified into four patterns according to the following definitions:

- Normal - FEV_1_/FVC ≥ 70 % and FEV_1_ > 80 % and FVC > 80 %
- Obstructed - FEV_1_/FVC < 70 % and FEV_1_ ≤ 80 %
- Restricted - FEV_1_/FVC ≥ 70 % and FVC ≤ 80 %
- Unclassified - all others

### Coronary heart disease history

Subjects from the OME population were asked if they had a history of a myocardial infarction, coronary artery stent, percutaneous transluminal coronary angioplasty (PTCA), or coronary artery bypass grafting. An affirmative response was considered positive evidence for self-reported coronary heart disease. Odds ratio for coronary heart disease was calculated by logistic regression.

### Survival

Date of death for subjects from the OME group was determined through a search of the social security death database as of May 2012. Subjects not appearing on the social security death database were assumed to be alive. An adjusted survival analysis (Cox Proportional Hazards regression) was run on the deceased subset. Data was adjusted for age at the time of spirometric testing, coronary heart disease, asthma, COPD and smoking status (current, former or never).

### Statistical measures

Participant characteristics were compared across spirometry groups (normal, obstructed, and restricted) using either chi-square tests of association or one-way Analysis of Variance (ANOVA), as appropriate. Sensitivity, specificity, positive and negative predictive values and ROC curve were determined. All analyses were completed using SAS version 9.2 (SAS Institute, Inc., Cary, NC). Bonferroni corrections were made for multiple statistical testing. A *p*-value of 0.05 or less was considered statistically significant.

### Availability of data and materials

According to the terms of approval of the Wake Forest University IRB, data can be published without written expressed consent from patients, if it is published in an aggregated form, without patient identifiers. However, if each patient’s data is to be shared publicly, there is the possibility of patient identification and would warrant a signed informed consent. Unique patient data is therefore not available in a publicly accessible repository.

## Results

### Prevalence of spirographic abnormalities when applying the 20/40 algorithm

**Fig. 1:**
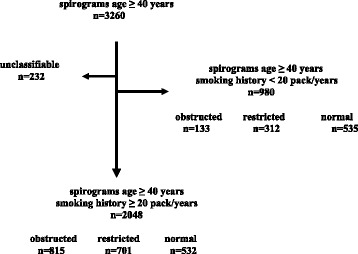
Of the 3260 spirograms evaluated in subject 40 years of age and older, 232 were unclassifiable. Spirographic pattern (obstructed, restricted or normal) is shown for the remaining 3028 grouped according to smoking history

In the OME group, spirograms were performed on individuals 40 years of age and older (*n* = 3260); 2222 individuals met algorithm criteria of ≥ 40 years of age and smoking history of at least 20 pack/years (Fig. 1). Of the 2222 spirograms that met ATS criteria for evaluation, 174 fell into the unclassified category. Of the remaining 2048 spirograms in this population, 815 (39.8 %) were consistent with an obstructed pattern, 701 (34.2 %) with a restrictive pattern, and 532 (26.0 %) were normal. Age, pack-years smoked, and body mass index (BMI) all differed significantly among the individuals in the three spirometric groups (Table 1). In general, the obstructed group was the oldest, had the highest rate of pack years of smoking, and was most likely to be current smokers with the restrictive group intermediate and the group with normal spirometry being the youngest and with the fewest pack years. The pattern for the BMI was slightly different with the highest average BMI in the restrictive group and the lowest in the obstructive group with those having normal spirometry intermediate. There was no significant difference in gender among the three groups, but men dominated the cohort by virtue of the method of recruitment.

**Table 1:** Subject characteristics – Occupational medical evaluation group

	Normal (*n* = 532)	Obstructed (*n* = 815)	Restricted (*n* = 701)	Significance
FEV_1_ % predicted [mean (SD)]	94.3 (8.8)	53.6 (16.3)	70.7 (11.6)	*P* < 0.001
FVC % predicted [mean (SD)]	92.4 (8.5)	70.4 (15.7)	67.7 (10.5)	*P* < 0.001
FEV_1_/FVC [mean (SD)]	77.0 (4.4)	56.3 (10.6)	78.3 (5.5)	*P* < 0.001
Gender [*n* (%) Male]	513 (99.4)	795 (97.6)	674 (96.2)	0.1646
Current smoker [*n* (%)]	129 (24.3)	269 (33.0)	189 (27.0)	*P* < 0.001
Obesity [*n* (%)]	191 (35.9)	211 (25.9)	339 (48.4)	*P* < 0.001
BMI [mean (SD)]	29.3 (4.4)	27.9 (5.0)	30.5 (5.1)	*P* < 0.001
Age [mean (SD)]	62.0 (9.1)	65.8 (8.5)	64.2 (9.2)	*P* < 0.001
Pack years [mean (SD)]	46.0 (24.9)	58.8 (30.7)	51.9 (27.8)	*P* < 0.001

### Self-reported coronary heart disease in subjects with abnormal spirometry (obstruction versus restriction)

The prevalence of self-reported coronary heart disease was greater (chi-square, *p* < 0.0002) in both the restricted (20.5 %) and obstructed (17.9 %) groups, compared to the normal spirometry group (12.0 %). Adjusting for BMI, smoking in pack years and age the odds ratio (OR) for having, self-reported coronary heart disease was significantly greater for individuals with restrictive (OR 1.70, 95 % confidence interval 1.23–2.35) and individuals with obstructive (OR1.39, 95 % confidence interval 1.00–1.93) spirometry patterns compared to those with normal spirometry. There was no significant difference in probability of coronary heart disease in the individuals with obstructive patterns compared to those with restrictive patterns, OR 1.22 (0.93–1.60).

### Survival of subjects with abnormal spirometry

Of the 2048 individuals available for analysis, e.g., those having obstructive or restrictive or normal spirometry patterns; 998 (48.7 %) died. Survival was significantly different between those with normal and abnormal spirometry patterns but not between those with the restrictive and the obstructive patterns. The hazard ratio (HR) for death for the restrictive pattern group (compared to the normal group) was 1.40, 95 % CI 1.08–1.72, indicating that the restricted group was at a 39.5 % higher risk of death than the normal group. Similarly, the HR for the obstructive pattern group was 1.53, 95 % CI 1.20–1.84 indicating a 52.7 % greater risk of death than the normal group.

### Symptoms and self-report of obstructive lung disease

There was a significant difference in reporting of respiratory symptoms among the three groups (Table 2). Subjects in the obstructive pattern group reported the highest rates of wheeze, cough, shortness of breath, and sputum production followed by those in the restrictive pattern group and then those with normal spirometry (*p* < 0.001).

**Table 2:** Self-report of symptoms and disease - occupational medical evaluation group

	Normal (*n* = 532)	Obstructed (*n* = 815)	Restricted (*n* = 701)	Significance
Wheeze [*n* (%)]	234 (44.0)	593 (72.8)	407 (58.1)	*p* < 0.001
Cough [*n* (%)]	285 (53.6)	587 (72.0)	414 (59.1)	*p* < 0.001
SOB [n (%)]	302 (56.8)	669 (82.1)	501 (71.5)	*p* < 0.001
Sputum [*n* (%)]	195 (36.7)	455 (55.8)	281 (40.1)	*p* < 0.001
# of Symptoms [mean (SD)]	1.9 (1.4)	2.8 (1.2)	2.3 (1.4)	*p* < 0.001
Self-reported COPD [*n* (%)]	32 (6.0)	304 (37.3)	77 (11.0)	*p* < 0.001
Self-reported asthma [*n* (%)]	25 (4.7)	135 (16.6)	43 (6.1)	*p* < 0.001
Self-reported asthma & COPD [*n* (%)]	3 (0.6)	77 (9.5)	14 (2.0)	*p* < 0.001
Self-reported CHD [*n* (%)]	64 (12.0)	146 (17.9)	144 (20.5)	*p* < 0.001

Based on self-report, among those patients with an obstructive spirometry pattern, 37.3 % had received a diagnosis of COPD alone with a further 9.5 % reporting a diagnosis of asthma and COPD, now labeled as ACOS (asthma-COPD overlap syndrome) [27] and 16.6 % reporting a diagnosis of asthma alone. A COPD diagnosis was also reported by 19.1 % of those with a restrictive spirometry pattern and by 11.3 % of those with a normal spirometry result.

### Sensitivity, specificity, positive and negative predictive value

The 20/40 rule was highly sensitive for obstructed spirometry with 86 % (95 % confidence interval 84–88 %) sensitivity (Table 3). Sensitivity of the algorithm for any spirometric abnormality (either obstructive or restrictive) was 77 % (confidence interval 75–79 %) The algorithm had a positive predictive value for any abnormality (either obstructive or restrictive) of 74 % (confidence interval 72–80 %).

**Table 3:** Sensitivity, specificity, positive and negative predictive values for OME group

	Sensitivity (%)	Specificity (%)	Positive predictive value (%)	Negative predictive value (%)	Disease prevalence (%)	Positive Likelihood Ratio	Negative Likelihood Ratio
For obstruction	85 (84–88)	41 (39–43)	40 (38–42)	86 (84–89)	31 (30–33)	1.45 (1.39–1.52)	0.34 (0.29–0.41)
For any spirometry abnormality (obstruction + restriction)	77 (75–79)	50 (47–53)	74 (72–80)	55 (51–58)	65 (63–66)	1.55 (1.45–1.65)	0.45 (0.41–0.50)

Values listed with (95 % confidence interval) OME - occupational medical evaluation

### 20/40 rule replication

There were 845 spirograms performed in the POC group. 98 failed to have three replicate loops greater than six seconds, and 74 were unclassifiable, based on our operational definitions, leaving 673 (79.6 %) for interpretation. Despite the addition of women in the POC group compared with the OME group, and a higher percent of current smokers, the percent of abnormal spirograms was similar in the POC group compared with the OME group (Table 4). Sixty-seven percent were abnormal, 38.3 % (258) with obstructive patterns, 28.4 % (191) with restrictive patterns, and 33.3 % (224) with normal spirometry.

**Table 4:** Subject characteristics – Algorithm replication population

	Normal *n* = 224	Obstructed *n* = 258	Restricted *n* = 191
FEV_1_ % predicted [mean (SD)]	94.84 (9.51)	56.20 (13.85)	70.52 (10.00)
FEV_1_ (L)(± SD)	2.79 (0.65)	1.73 (0.56)	2.14 (0.57)
Sex (% male vs. female)	50 vs. 50	71 vs. 29	52 vs. 48
Smoke Status (Current vs. Past)	58 vs. 42	65 vs. 35	58 vs. 42
BMI [mean (SD)]	30.06 (6.75)	27.01 (6.25)	31.65 (7.19)
Age [mean (SD)]	59.90 (9.68)	65.41 (10.73)	61.09 (9.60)
Pack years [mean (SD)]	48.10 (43.89)	59.55 (36.81)	50.90 (46.88)

## Discussion

### Main findings

Using a simple 20/40 rule based solely on age and smoking history identifies a high percent of individuals (74 % in the initial population and 67 % in the POC group) with lung function abnormalities (both obstructive and restrictive spirometry patterns). Further those meeting the 20/40 rule who have lung function abnormalities have an increased risk of all-cause mortality and a high rate of reported coronary heart disease, compared to individuals with normal spirometry.

Using the simple 20/40 rule in primary care could facilitate the appropriate use of spirometry evaluation and support the diagnosis of COPD in up to 40 % of patients with limited need for further testing. This spirometry testing could be accomplished in the primary care office [28–30] allowing rapid diagnosis and initiation of appropriate COPD management. For the 30 % of individuals with a restrictive lung pattern, the addition of lung volumes and perhaps other elements of full pulmonary function testing may be required to determine the accuracy of the restrictive pattern and assess possible associated diagnoses.

Our evaluation of the 20/40 rule confirmed the previously reported over and under diagnosis of COPD within this population. Of those who remembered receiving a COPD or COPD plus asthma diagnosis, 11.3 % had normal spirometry assessment suggesting that a different condition may be causing their symptoms—potential over diagnosis. Conversely, 53.2 % of the individuals with an obstructive pattern did not believe they had received a COPD diagnosis (under diagnosis) likely prohibiting them from receiving any of the therapies known to improve symptom burden or prolong life [31].

### Relationship to the literature

Use of the 20/40 rule goes beyond simply “screening” for risk of COPD and includes the opportunity to evaluate for the presence of restrictive spirometry patterns which may also be seen with COPD or with other conditions in the lungs and other organ systems. Spirometric results from our study groups provide important new data on rates of restrictive spirometry patterns in long-term smokers. Published values for prevalence of a restricted pattern on spirometry in unselected populations vary from 10.3 to 12.3 % [8, 32]. Data from the Multi-Ethnic Study of Atherosclerosis (MESA) [33] showed that among smokers the prevalence of a restricted pattern on spirometry was 10 %, increasing by 8 % (95 % confidence interval 3–12 %) for each 10 pack-years smoked. The prevalence of a restricted pattern in MESA was 16 % for the > 20 pack year cohort, who had a smoking history significantly lower than our OME group (36 pack years, 95 % CI 27–50 vs 51.9 ± 27.8 pack years). Allowing for the additional increase in prevalence of restriction for each pack year smoked found in MESA, the 28.4 and 34.2 % prevalence of restrictive spirometry in our study populations is consistent with the MESA data and provides a useful population estimate in longer-term smokers aged 40 years and older.

Under recognition of lung function abnormalities in smokers is common. But most studies focus on the airflow obstruction reported to occur in up to 50 % of long-term smokers [34] for which under recognition is well documented [4, 16, 17]. However, the under-recognition of a restricted pattern in smokers is less well studied, but is clearly evident from our results which also highlights the potential consequences of under-recognition of both obstructive and restrictive lung function abnormalities. With airflow obstruction, these consequences include under treatment, that may lead to increased exacerbation frequency, symptoms, decreased quality of life, and increased medical costs [35]. Restrictive spirometry patterns are also seen in smoking related conditions such as fibrotic interstitial lung disease, (RBILD or respiratory bronchiolitis with interstitial lung disease) [10, 36] muscle weakness [11], heart disease [12], obesity and the metabolic syndrome [13]. A restricted spirometry pattern has been reported to be associated with numerous cigarette and non-cigarette related co-morbidities, including hypertension, type II diabetes, atherosclerosis, cardiovascular disease, and all-cause mortality, in a manner that is statistically independent of confounding variables, such as diabetes, obesity, and smoking history [37–47]. Our data shows that the prevalence of coronary heart disease and all-cause mortality is significantly greater for smokers age ≥ 40 years with a restricted pattern than for those with normal spirometry. In fact, there was no significant difference in coronary heart disease prevalence and all-cause mortality, between subjects with an obstructed and those with a restricted pattern on spirometry. Under-recognition of both obstruction and restriction in smokers, age ≥ 40 years, is a common occurrence with significant health consequences.

Other approaches have been developed for selecting patients at high risk of airflow limitation. These include use of questionnaires [48–52], risk prediction models [53] and handheld flow meters [54, 55]. Most questionnaires developed for COPD are related to patient outcomes of quality of life. The COPD population screener (COPD-PS) is a 5 question tool with a positive predictive value of 56.8 % and negative predictive value of 86.4 % [50]. van Schayck et al. [52] and Calverley et al. [56] developed a population-based screening questionnaire for COPD using NHANES III data. Price and coworkers [51] published an 8-item COPD questionnaire in patients with a positive smoking history that included items related to age group, body mass index, pack-year history, and symptoms. Finally, Freeman and colleagues [57] utilized age, cough, dyspnea, and wheezing in a questionnaire to identify patients with COPD in a primary care setting who had a positive smoking history, history of respiratory medication use, or of asthma. The COPD-PS differs from these previous questionnaires because it can be used regardless of history of respiratory problems or smoking and it contains a disease-impact item. Population screeners when used in combination with peak expiratory flow measurement (PEF) however have been shown to add little to PEF alone [54, 58, 59]. A new questionnaire developed by the High Risk-COPD Screening Group is currently undergoing testing in combination with PEF [60]. Preliminary data is impressive [61]. A single published risk prediction model utilizing sex, socioeconomic status and previously recorded asthma diagnosis has performed well in an initial derivation cohort of 480,903 and validation cohort of 247,755 subjects with area under the receiver operating curve of 0.8 [53].

### Strengths and limitations

Limitations in this study include the use of a fixed threshold cut-off for the definition of obstruction (FEV_1_/FVC < 70 %). This fixed ratio threshold has been demonstrated to introduce age related bias [62]. Use of a fixed ratio can result in misclassification in more than 1/4^th^ of tests [63] and over diagnosis of airflow obstruction in older subjects [64]. However, the further qualification of FEV_1_ < 80 % placed on our obstruction definition reduces over diagnosis significantly [65]. FVC and FEV_1_ were assessed before bronchodilator, allowing the possibility that a portion of the obstructed spirometries might display reversibility, suggestive of a purely asthmatic component, rather than chronic obstructive pulmonary disease [66].

### Implications for policy, practice and research

The goal of this study was to increase use of spirometry in the primary care setting. Only half of primary care providers routinely use spirometry in their practice of medicine [67]. Among primary care physicians spirometry use was associated with agreeing that the data are necessary for accurate diagnosis, and believing that they were trained to perform and interpret the test [68]. In view of these reports and others, we elected to use a simple rule that might increase use of spirometry in the primary care setting without significantly sacrificing accuracy. We wanted to show that use of this *simple* rule could enhance diagnostic yield that had a clinical impact such as increased risk for CHD and death. Furthermore, it distinguished individuals that would benefit from lung volume measurement. Using the 20/40 rule resulted in detection of an abnormality on spirometry in nearly 70 % of individuals tested.

## Conclusion

We show this simple 20/40 rule, based solely on age and smoking history performs well in predicting pulmonary function abnormalities. Sensitivity of the algorithm was 85 % for an obstructive pattern and 77 % for any abnormality on spirometry. Positive and negative predictive values for any spirometric abnormality were 74 and 55 %, respectively. Furthermore, we show that pulmonary function abnormalities, which are often under-recognized, are associated with coronary heart disease and all-cause mortality and might prompt further valuable clinical evaluations. Using an easily applied 20/40 rule based on information that should be available in all medical records, age 40 years or greater and 20 or more years of smoking can simplify the decision of when and with whom to complete spirometry. The results provide clinically significant information for the diagnosis of not only COPD but identification of restrictive lung patterns both of which are associated with increased mortality risk. The 20/40 rule is a simple, sensitive, predictor of lung function abnormalities that are markers for COPD, coronary heart disease and all-cause mortality.

Under diagnosis of chronic lung disease is common. NHANES estimates that >50 % of individuals with airflow obstruction are undiagnosed [4]. Preliminary data suggests that diagnosis of chronic lung disease is linked to perception and report of symptoms. In a population of screened individuals, matched for FEV1, subjects with a diagnosis of COPD had greater symptom scores than those previously unrecognized as COPD [69]. Furthermore, subjects with unrecognized COPD experienced a greater number of postoperative respiratory complications, than subjects diagnosed with COPD, despite similar lung function [70]. These data would suggest that under recognition of symptoms leads to under diagnosis of chronic lung disease. But under recognition of symptoms does not protect patients from the common respiratory complications of surgery that their compromised lung function causes, thus, making a case for selective use of spirometry based on age and smoking history.

## Abbreviations

ACOS, asthma-COPD overlap syndrome; ANOVA, Analysis of Variance; BMI, Body mass index; CI, Confidence interval; COPD, Chronic obstructive pulmonary disease; FEV_1_, Forced expiratory volume in one second; FVC, Forced vital capacity; GOLD, Global initiative for chronic obstructive lung disease; HR, Hazard ratio; IRB, Wake Forest University Institutional Review Boards; MESA, Multi-Ethnic Study of Atherosclerosis; NHANES, National Health and Nutrition Examination Survey; NICE, National Institute for Clinical Excellence; OR, Odds ratio; PTCA, Percutaneous transluminal coronary angioplasty; RBILD, respiratory bronchiolitis with interstitial lung disease; SE, Standard error
